# Enhanced mass transfer of pulsed vacuum pressure pickling and changes in quality of sour bamboo shoots

**DOI:** 10.3389/fmicb.2022.981807

**Published:** 2022-09-16

**Authors:** Jian-Wu Dai, Qing Zhang, Ming Li, Lian-Jie Li, Li-Jia Xu, Yao-Wen Liu, Peng-Fei Yin, Shu-Xiang Liu, Yong-Peng Zhao, Kai-Yun Gou, Ying-Lu Li, Wen Qin

**Affiliations:** ^1^College of Mechanical and Electrical Engineering, Sichuan Agricultural University, Ya'an, China; ^2^College of Food Science, Sichuan Agricultural University, Ya'an, China; ^3^College of Science, Sichuan Agricultural University, Ya'an, China

**Keywords:** sour bamboo shoots, pulsed vacuum pressure pickling, salt distribution, pH value, LAB content

## Abstract

Sour bamboo shoot is a traditional Chinese fermented vegetable food. The traditional pickling method of sour bamboo shoots has the disadvantages of being time-consuming, inhomogeneous, and difficult to control. Pulsed vacuum pressure pickling (PVPP) technology uses pulsed vacuum pressure to enhance the pickling efficiency significantly. To demonstrate the effects of salt content and PVPP technical parameters on the fermentation of bamboo shoots, the sample salinity, pH value, color, crunchiness and chewiness, nitrite content, and lactic acid bacteria content during the pickling process were investigated. The salt content inside the bamboo shoots gradually increased to the equilibrium point during the pickling process. The pickling efficiency of bamboo shoots under PVPP technology increased by 34.1% compared to the traditional control groups. Meanwhile, the uniform salt distribution under PVPP technology also obtained better performance in comparison with the traditional groups. The pH value declined slowly from 5.96 to 3.70 with the extension of pickling time and sour flavor accumulated progressively. No significant differences were found in the color values (L^*^, a^*^, and b^*^) and the crunchiness of the bamboo shoot under different salt solution concentrations, vacuum pressure, and pulsation frequency ratio conditions. Colony-forming unit of lactic acid bacteria (CFU of LAB) decreased, to begin with, and then increased until the 6th day, followed by a declining trend in volatility. The nitrate content of bamboo shoots samples under PVPP treatments did not exceed the safety standard (<20 mg/kg) during the whole fermentation process, which proves the safety of PVPP technology. In conclusion, PVPP technology can safely replace the traditional method with better quality performance. The optimal PVPP processing conditions (vacuum pressure 60 kPa, 10 min vacuum pressure time vs. 4 min atmospheric pressure time, salt solution concentration 6%) have been recommended for pickling bamboo shoots with high product quality.

## Introduction

Sour bamboo shoot is considered a traditional local flavor food in China, which has the desirable characteristics of crispy taste and sour flavor. According to NBS(National Bureau of Statistics)of China,the total annual production of bamboo shoots in China can reach up to 1.03 million tons, and its total output value exceeds 21.7 million dollars (National Bureau of Statistics of China, 2020). The key step in the processing of sour bamboo shoots is to ferment them by completely submerging the bamboo shoots in a salt solution for a certain period of time (Xiao et al., 2018). During the pickling process, the bamboo shoots gradually get sour due to lactobacillus activity (Liu et al., 2019). Mass transfer of salt content from the external solution to the interior layers of the bamboo shoots occurs simultaneously.

Currently, the traditional atmospheric absorption method, treated as the main pickling way, is to immerse the bamboo shoots in the pickling salt solution directly under atmospheric pressure. It usually takes about 10–20 days for the shoots to ferment at around 22°C before it becomes ready to eat (Cao et al., 2017; Wang et al., 2020a; Fan et al., 2022). The low pickling efficiency limits and hinders the industrial production of sour bamboo shoots. And the excessive marinating time is also likely to cause the microorganism content in the sour bamboo shoots to exceed the safety standards (Zhang et al., 2015a; Liang et al., 2020). Therefore, pickling efficiency and microorganism content are two main factors affecting the pickling quality of sour bamboo shoots. However, given the low pickling efficiency and safety standardization, choosing an appropriate processing technology for the industrial production of sour bamboo shoots is still an urgent problem to be solved.

Pulsed vacuum pressure pickling (PVPP) technology is an efficient pickling technique with about 10 times higher than the traditional atmospheric immersion method in pickling efficiency based on recent findings (Chen and Gao, 2006; Wang and Gao, 2010). The PVPP operating procedure includes adjusting the on-off time of the vacuum pump to ensure the pressure in the marinating jar is kept in a vacuum environment for a longer duration followed by normal pressure for a shorter duration and in continuous iterations. Previous research findings suggest that one possible reason for the high pickling efficiency is the formation of pressure gradients between the inner and outer layer of the bamboo shoots during the PVPP process, which improves the structure's permeability and consequently promotes the rapid infiltration of salt (Wang et al., 2010).

Based on the above analysis, the main objectives of this research are: (a) to investigate the impact of different salt solution concentrations, pulsation frequency ratio, and vacuum pressure in the vessel chamber on the pickling process kinetics and overall rating of sour bamboo shoots, (b) to explore the salt distribution and lactic acid bacteria content variation dynamics of sour bamboo shoot slices using PVPP technology, (c) to optimize the processing parameters of sour bamboo shoots to provide a certain theoretical reference for the industrial production of sour bamboo shoots.

## Materials and methods

### Raw material

Fresh bamboo shoots were purchased from a local farm market in Ya'an, Sichuan province, China (29°58′51″N, 102°56′57″E). The samples with uniform appearance (radius of 25 ± 2 mm and weight of 100 ± 3 g) were selected to ensure consistent physical properties and then stored at 4°C and 90% relative humidity before formal experiments. The initial moisture content of the bamboo shoots was 10.57 kg·kg^−1^ (d.b.), which was recorded using the Association of Official Analytical Chemists method no. 934.06 (AOAC, 2005).

### Equipment

**Figure 1:**
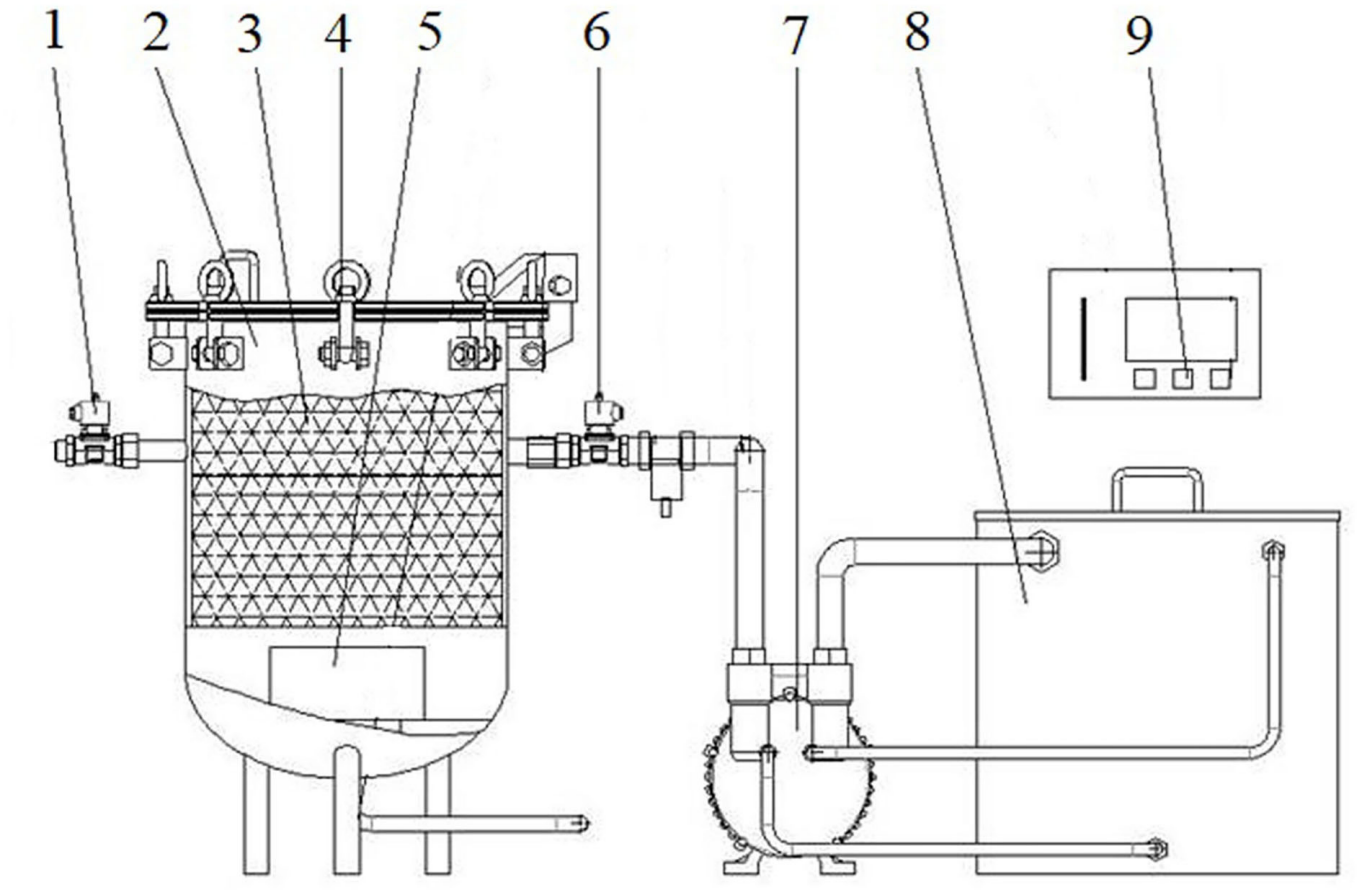
Structure diagram of pulsed vacuum pressure pickling device. 1. Air solenoid valve, 2. Pressure vessel, 3. Material box, 4. Lifting bolt, 5. Pickling solution uniform system, 6. Air extraction solenoid valve, 7. Vacuum pump, 8. Cooling water tank, 9. Control system.

The structure diagram of the pulsed vacuum pressure pickling device for bamboo shoots is shown in Figure 1. This equipment mainly consists of a stainless-steel pickling tank, vacuum pump, and PID control system. The cylindrical tank has an inner diameter of 0.3 m, a height of 0.4 m, and an effective volume of ~30 L. Proportional-Integral-Derivative (PID) controller AI-207 (Yudian, Xiamen, China) is used to regulate chamber pressure in constant pulsating cycles between the atmospheric pressure and vacuum pressure in their respective holding time. During the pickling experiment, the chamber pressure was regulated between 40 and 101 kPa by operating a vacuum pump 2BV5 (Boshan, Zibo, China). The vacuum pressure holding time and atmospheric pressure holding time varied from 5 to 15 min, and 3–5 min, respectively.

### Single factor experimental design

The fresh bamboo shoot samples were peeled and cut into halves lengthwise. Then the samples were immersed in the chamber without blanching and pickled under different PVPP conditions. In preliminary experiments, it was found that the bamboo shoots would appear brownish in color when the pulsation frequency ratio was kept below 5:4 min/min, and the pickling process of sour bamboo shoots would be much longer if the pulsation frequency ratio exceeded 20:4 min/min. Additionally, according to the FAO, a high concentration of salt is detrimental to people's health. Therefore, considering these parameters and the limitation of the PVPP equipment, different salt solution concentrations (0, 3, 6, and 9%), PVPP vacuum pressures (20, 40, 60, and 80 kPa), and PVPP pulsation frequency ratios (5:4, 10:4, 15:4, and 20:4 min/min) were used to investigate the salt content and pickling quality of sour bamboo shoots adopting the single factor experiment method as presented in Table 1. The sample weight of each group was kept at 200.0 ± 2.0 g for all runs. Few reports (Fan et al., 2015) suggest blanching as a pretreatment procedure in sour bamboo shoots pickling for color protection. Therefore, bamboo shoots immersed in boiled water for 15 min were treated as blanched samples. In this study, blanched and unblanched bamboo shoots under the atmospheric impregnation method were both treated as the traditional control experimental groups. The quality attributes of the samples were recorded every 2 days until the marinating process was completed.

**Table 1:** Single factor experiment design with run conditions included.

**No**.	**Treatment group**	**Salt solution concentration**	**Vacuum pressure**	**Pulsation frequency ratios**
1	Unblanched with different salt solution concentrations	0%	Atmospheric pressure	/
2		3%		
3		6%		
4		9%		
5	Blanched with different salt solution concentrations	0%	Atmospheric pressure	/
6		3%		
7		6%		
8		9%		
9	Different vacuum pressures	6%	20 kPa	15:04 min/min
10			40 kPa	
11			60 kPa	
12			80 kPa	
13	Different pulsation frequency ratios	6%	60 kPa	5:04 min/min
14				10:04 min/min
15				15:04 min/min
16				20:04 min/min

### Quality factor evaluation

#### Sample salinity

The sample salinity content is measured through a salinity meter WS-500 (Xudu Co. Ltd, Nanjing, China) with an accuracy of ±0.3%. The bamboo shoot samples were cut into two parts, the inner layer and the outer layer, along the thickness. Each part was pounded into a paste and placed on the prism to measure the value. The digital LCD panel displays the reading directly, avoiding subjective and wrong numerical interpretation.

The uniformity in the salt distribution in the bamboo shoots during the pickling process can be expressed by *K* as follows (Gong et al., 2021):


(1) $$\begin{array}{r} K=\frac{\sigma_{\mu }}{\mu }\times 100\% \end{array}$$

where *K* is the non-uniform coefficient of salt distribution in each group. The smaller the coefficient, the better the salt distribution uniformity. σ_μ_ represents the standard deviation, where μ is the mean value of each experimental group.

#### pH value

For pH analysis, 10 g homogenate of bamboo shoots samples was first diluted with 90 mL deionized water. Then a PHB-4 acidity meter (Lichen, Shanghai, China) was calibrated with a standard buffer solution and used to measure the sample pH value (Liang et al., 2020; Rao et al., 2020). All testing procedures were repeated three times and the average value was taken for further analysis.

#### Color measurement

The color attributes of fresh and pickled bamboo shoots were collected by a colorimeter NR-10QC (3NH, Shanghai, China) five times. After removing the maximum and minimum values, the mean value of the remaining data was obtained and used for calculation. Total color difference (E^*^) was obtained through the following equation (Dai et al., 2020; Deng et al., 2020; Tofalo et al., 2021):


(2) $$\Delta E^{*}=\sqrt{{\left(L^{*}-L_{0}\right)}^{2}+\;{\left(a^{*}-a_{0}\right)}^{2}+\;{\left(b^{*}-b_{0}\right)}^{2}}$$

where L^*^, a^*^, and b^*^ are the lightness, redness/greenness, and yellowness/blueness of sour bamboo shoots; *L*_0_, α_0_, and *b*_0_ represent the control values of fresh bamboo shoots, respectively.

#### Nitrite content

The determination of residual nitrite content inside sour bamboo shoots is obtained based on the nitrite colorimetric method AOAC 973.31 (AOAC, 2005). A 5 g bamboo shoot sample was fully mashed with distilled water and stabilized until its capacity reached 100 mL. Then, 4 mL of the supernatant was taken as the blended solution, to which 5 drops of nitrite reagent were added and maintained at room temperature for 10 min. The nitrite tester YXSY-1A (Haifeng Co. Ltd, Shanghai, China) with a standard colorimetric card was used to obtain the residual nitrite content and expressed as mg NaNO_2_/kg of the sample (mg NaNO_2_/kg sample).

#### Lactic acid bacteria (LAB) measurement

The methodology for counting lactic acid bacteria content is assessed in triplicates using the method of Rao et al. (2019) with partial modifications. After diluting with 0.9% saline solution, LAB counts in different groups were determined by enumeration on MRS agar (Tuopu Biol-Engineering Co., Shangdong, China) and incubated at 37°C for 48 h. Lactic acid bacteria content is expressed as CFU per milliliter.

#### Texture profile analysis

Crunchiness and chewiness are related to the structural properties of the bamboo shoots and important for evaluating the texture quality of the pickled products. Texture analyzer TA.1 (Baoman, Suzhou, China) was used to measure the sample's crunchy and chewy values. Samples with the same size and shape were selected and put on the texture analyzer for puncture tests. The condition parameters were set as follows (Dai et al., 2019): probe model of TA/2N, speed before measurement of 3.0 mm/s, speed after measurement of 3.0 mm/s, and test speed of 2.0 mm/s, respectively. Analysis of crunchiness (N) and chewiness (N) was performed in triplicate for all treatments.

### Comprehensive score

The standardization of each evaluation index is calculated according to Eq. (3). The optimal value of each index was set between 10 (maximum value) and 1 (least value) (Wang et al., 2020b).


(3) $$\begin{array}{r} d_{i}=\frac{X_{\max}-X_{i}}{X_{\max}-X_{\min}} \end{array}$$

where *d*_*i*_ is the normalized value of indicators, *X*_*i*_ is the actual measured value, *and X*_*max*_ and *X*_*min*_ represent the maximum and the minimum index values, respectively. Salt content, crunchiness, chewiness, and L^*^ value of bamboo shoots were categorized as positive indicators, which meant the higher the indicator value, the better the sample quality. And pH value and Δ*E* were categorized as negative indicators, which had the opposite assessment.

Each index weight coefficient (*w*_*j*_) was obtained based on the entropy weight method (Jing et al., 2020):


(4) $$\begin{array}{r} w_{j}=\frac{1-E_{j}}{n-\sum_{j=1}^{n}E_{j}} \end{array}$$

where *E*_*j*_ is comentropy, ${E_{j}=-{\left(\ln\;m\right)}^{-1}\sum }_{i=1}^{m}P_{ij}\ln\;P_{ij}$; *n* is the number of influencing factors.

The comprehensive score of the pickled bamboo shoots under different pickling parameters was obtained based on Eq. (5) (Zhang et al., 2015b):


(5) $$\begin{array}{r} F=d_{1}w_{1}+d_{2}w_{2}+d_{3}w_{3}+d_{4}w_{4}+\;d_{5}w_{5}+\;d_{6}w_{6} \end{array}$$

where *F* is the comprehensive score; *d*_1_, *d*_2_, *d*_3_, *d*_4_, *d*_5_, and *d*_6_ represent the standardized values of salt content, pH value, crunchiness, chewiness, L^*^ and *C* values of the pickled bamboo shoots, respectively; *w*_1_, *w*_2_, *w*_3_, *w*_4_, *w*_5_, and *w*_6_ are the related weight coefficient of evaluation index.

### Statistical analysis

Each group of experiments was carried out in triplicate and the mean value was calculated for drawing the curves and further data analysis. SPSS 17.0 statistical software was used to determine the data significance by ANOVA at a *p* = 5% probability level.

## Results and discussion

### Effects of PVPP parameters on salt content

**Figure 2:**
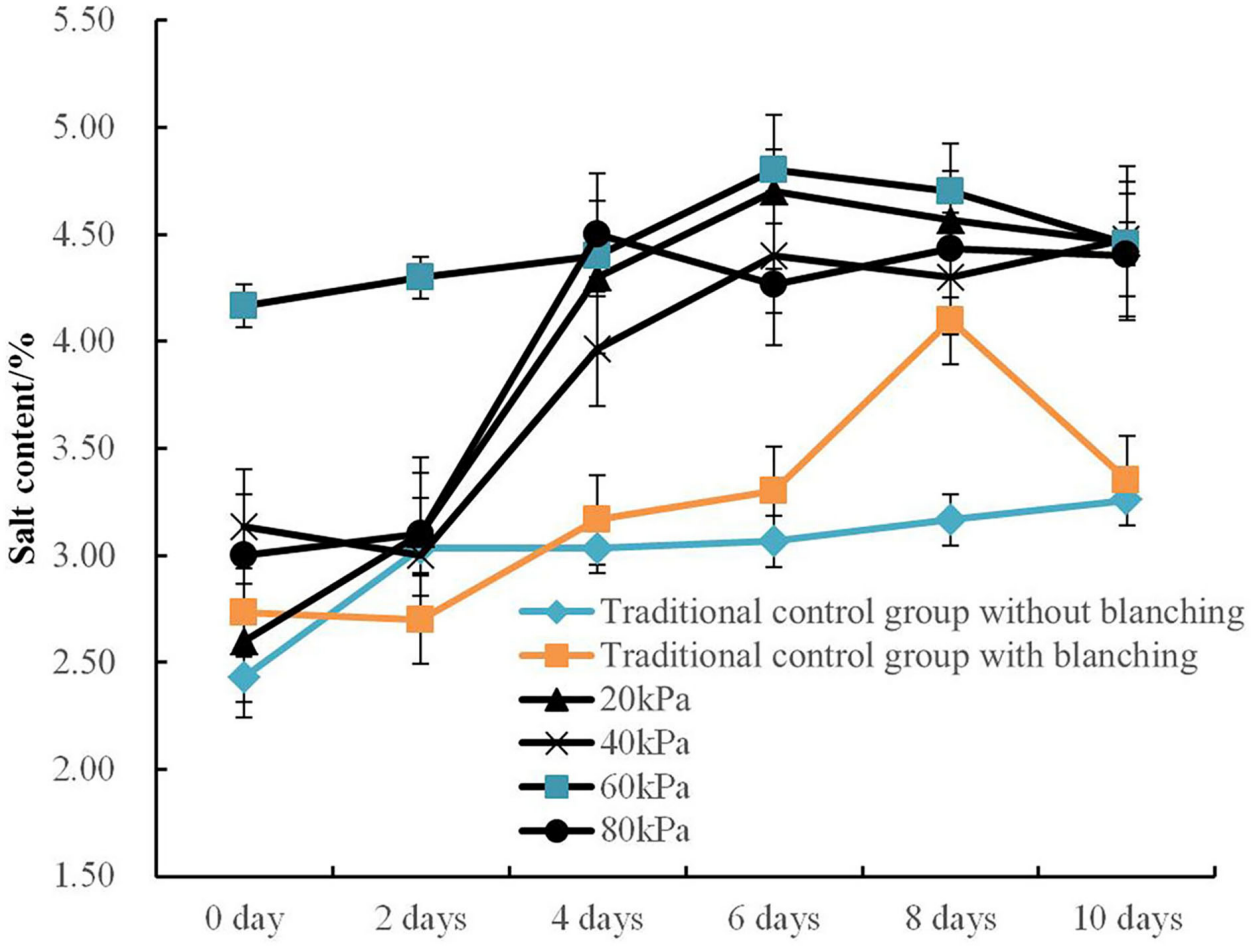
Variation kinetic curves of salt content in PVPP groups with different vacuum pressures.

**Figure 3:**
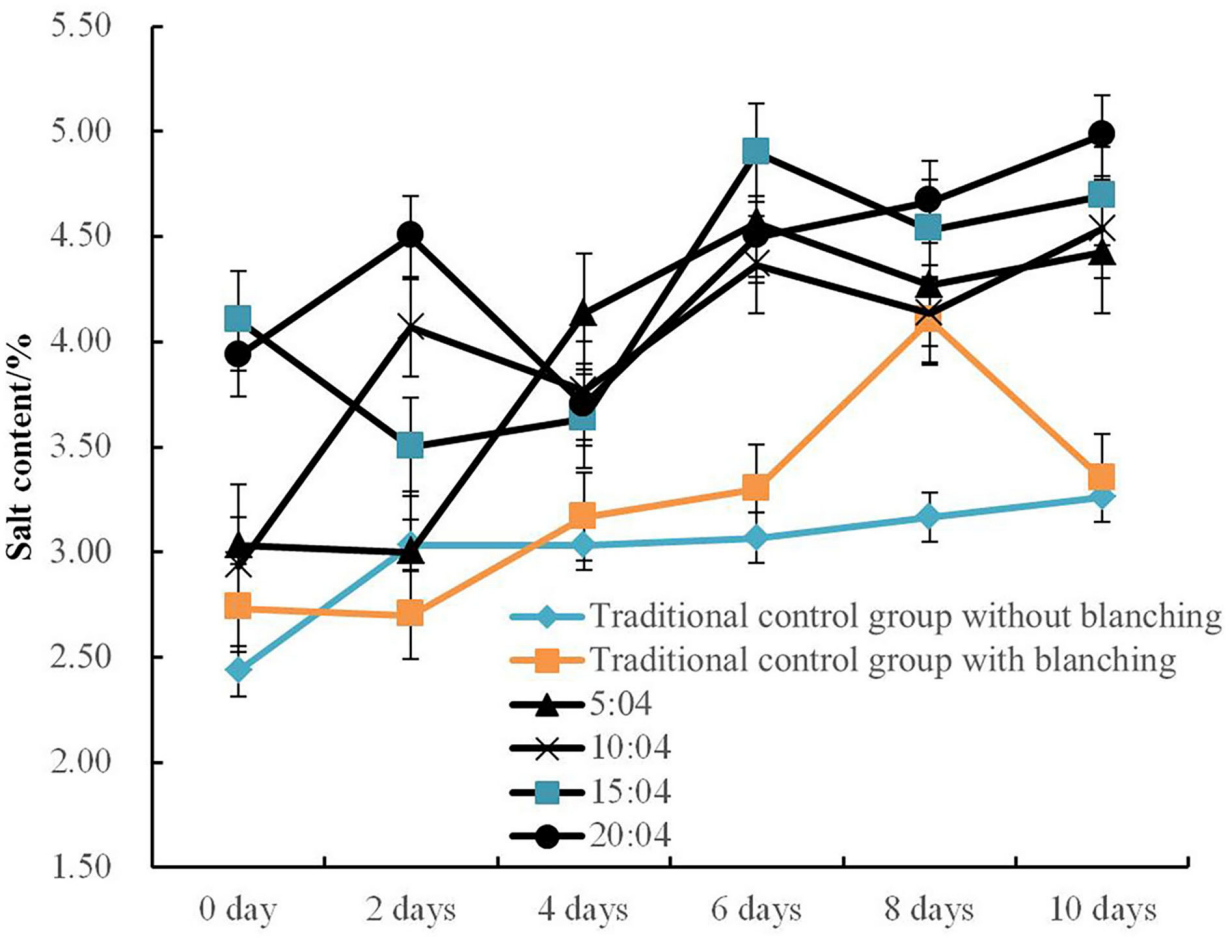
Variation kinetic curves of salt content in PVPP groups with different pulsation frequency ratios.

The impacts of different vacuum pressure and pulsation frequency ratios on salt changes and salt distribution in bamboo shoots compared with the traditional atmospheric method under the same salt solution concentration of 6% is shown in Figures 2, 3.

#### Salt changes

The influence of different vacuum pressure (20, 40, 60, and 80 kPa) under PVPP technology on the pickling efficiency and salt content of bamboo shoots with the same pulsation frequency ratio of 15:04 min/min are illustrated in Figure 2.

It can be seen that the salt content of bamboo shoots using PVPP technology is significantly higher than the bamboo shoots pickled using the traditional method, demonstrating that PVPP technology could significantly enhance pickling efficiency. The overall variation of salt content in PVPP groups under different vacuum pressures reveals that the salt content in bamboo shoots gradually reaches an equilibrium level of 4.43% with the extension of pickling time, while the traditional control group without blanching shows a similar trend with a much lower equilibrium level of 3.28%. The promotion of salt content in bamboo shoots during the PVPP process is due to the internal and external pressure difference caused by PVPP technology and therefore enlarges the pore sizes and structure permeability (Wang et al., 2010, 2018). The traditional control group with blanching also revealed obvious peaks on the 8th pickling day. Yang et al. (2021) find this variation tendency similar to the salinity changes curves of Sichuan paocai during fermentation.

The salt content inside the bamboo shoots under different pulsation frequency ratios (5:4, 10:4, 15:4, and 20:4 min/min) begin to gyrate up with fluctuations as the pickling days extend (Figure 3). The highest salt content of samples in PVPP groups during the pickling process varied from 4.40 to 4.97%, and it was about 34.1% higher than that of traditional control groups. Wang et al. (2013) observed that adjusting the pulsation frequency ratio could expand the pore size of the shell and significantly enhance the mass transfer efficiency in the egg pickling process.

#### Salt distribution

Uniformity in the salt distribution in the bamboo shoots under different salt solution concentrations, vacuum pressure, and pulsation frequency ratio are listed in Table 2.

**Table 2:** Salt distribution uniformity of bamboo shoots between outer layer and inner layer under different technical parameters.

**Technical parameters**	**Outer layer of bamboo shoots (%)**	**Inner of bamboo shoots (%)**	**Salt distribution uniformity**
0%	0.00	0.00	–
3%	2.26 ± 0.05^h^	2.01 ± 0.01^h^	0.18
6%	3.42 ± 0.07^g^	3.26 ± 0.07^g^	0.12
9%	5.06 ± 0.06^ab^	4.96 ± 0.05^b^	0.07
Blanched 0%	0.00	0.00	–
Blanched 3%	2.01 ± 0.01^i^	1.95 ± 0.07^h^	0.04
Blanched 6%	3.35 ± 0.05^f^	3.39 ± 0.08^g^	0.03
Blanched 9%	5.09 ± 0.07^a^	5.10 ± 0.07^a^	0.01
20 kPa	4.46 ± 0.06^de^	4.26 ± 0.01^f^	0.14
40 kPa	4.48 ± 0.07^de^	4.33 ± 0.02^e^	0.11
60 kPa	4.46 ± 0.05^de^	4.32 ± 0.02^f^	0.10
80 kPa	4.40 ± 0.01^e^	4.41 ± 0.05^de^	0.01
5:04 min/min	4.42 ± 0.02^e^	4.38 ± 0.06^de^	0.03
10:04 min/min	4.54 ± 0.01^d^	4.44 ± 0.01^d^	0.07
15:04 min/min	4.69 ± 0.04^c^	4.56 ± 0.02^c^	0.09
20:04 min/min	4.78 ± 0.07^b^	4.57 ± 0.07^c^	0.15

Different letters in the same column reveal significant difference at α = 5% level according to the Duncan test.

As the concentration of the salt solution increases, the uniformity in salt distribution inside the shoots gradually converges, and the data dispersion degree constantly decreases. Bamboo shoots with blanching pretreatment showed a better distributional uniformity than unblanched samples, probably because the blanching treatment destroys the cellular structure inside the bamboo shoots and improves tissue permeability. Hence it reduces the resistance to the pickling process and improves the salt distribution uniformity in different positions of the bamboo shoots (Wang et al., 2017). Compared with the traditional control group without blanching at the same salt concentration of 6%, salt content inside and outside of bamboo shoots under different PVPP parameters had a relatively smaller coefficient, suggesting that PVPP technology is beneficial for the uniform distribution of salt content. This might be because, during the PVPP process, the salt content diffuses from the salt solution to the inside of bamboo shoots under vacuum pressure, while it inverses the mass transfer diffusion from the inner layer of bamboo shoots to the external solution under atmospheric pressure due to the pressure gradients. This phenomenon leads to bidirectional mass transfer of salt content inside the bamboo shoots and improves salt distribution uniformity (Wang et al., 2013). The optimum uniformity in the distribution of salt content was obtained at the salt solution concentration of 6% under the PVPP vacuum pressure of 80 kPa and pulsation frequency ratio of 15:04 min/min.

### pH value of bamboo shoots during pickling

**Figure 4:**
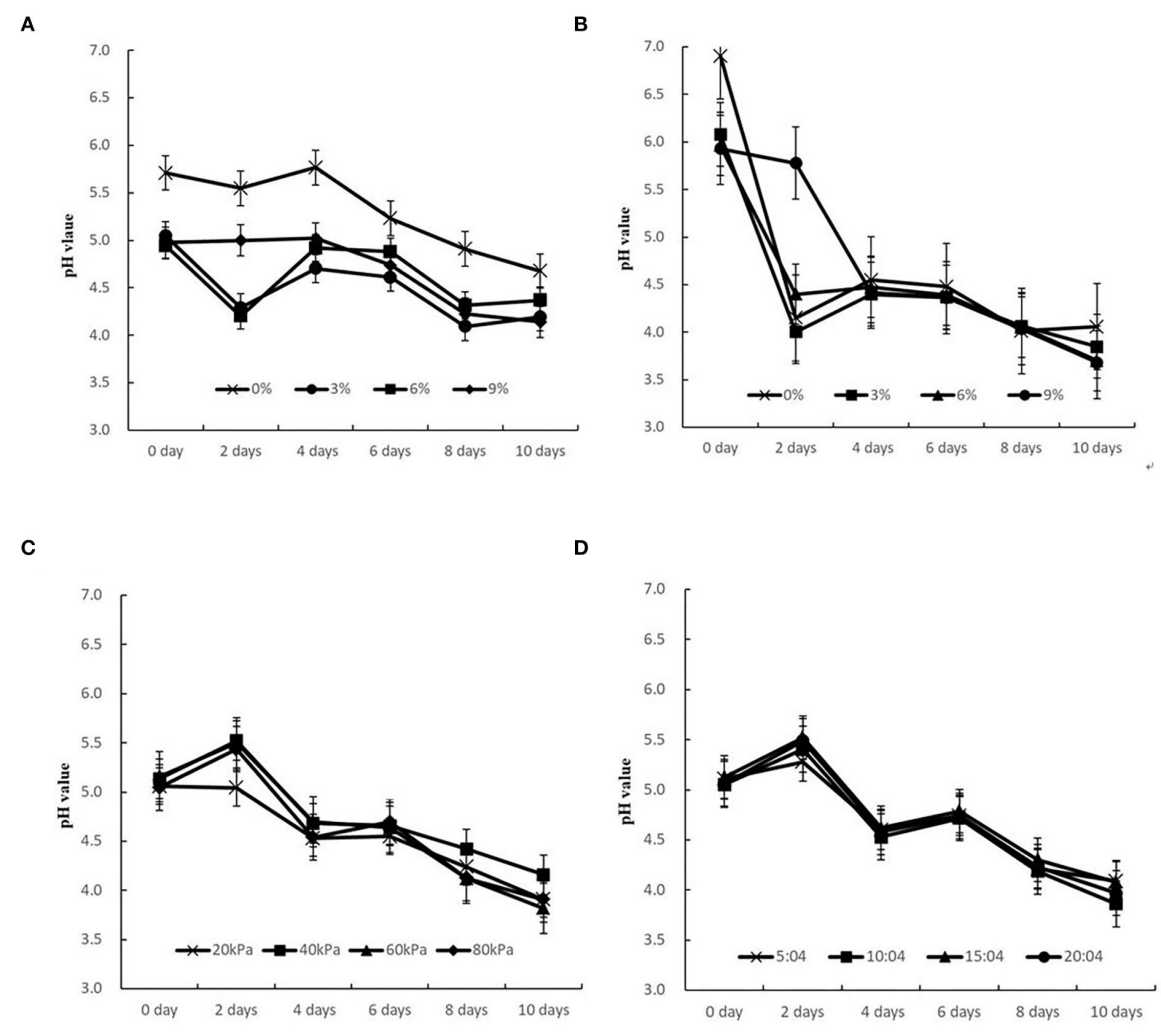
pH change dynamic curves of sour bamboo shoots under different technical conditions. **(A)** Traditional control groups without blanching under different salt concentrations, **(B)** Traditional control groups with blanching under different concentrations, **(C)** PVPP groups with different vacuum pressures **(D)** PVPP groups with different pulsation frequency ratios.

The pH values of bamboo shoots under different PVPP parameters are drawn as kinetics curves. With prolonged marinating duration, all PVPP groups show a downward trend in the overall changes (Figure 4), except for a slight rise on the 2nd day, indicating that the acidity of the samples increases gradually. While the bamboo shoots are being fermented, the reproduction and metabolism of microorganisms continue which enhances the accumulation of various organic acids of metabolites and the pH value drops continuously (Fan et al., 2015). It can be found that the pH value in the traditional control group without blanching shows an overall downward trend with an increase in the concentration of the salt solution. However, this trend was no longer obvious when the salt solution concentration exceeds 3% (Figure 4A). This could be because the optimum salt concentration for the growth of lactic acid bacteria is ~4.5%. When the salt concentration increases to 6 and 9%, the high osmotic pressure of the solution inhibits the proliferation of lactic acid bacteria, thereby affecting the metabolite content and pH value (Li et al., 2022; Ou et al., 2022). The blanched control group obtained a more sour quality being marinated for the same duration in comparison with the traditional unblanched control group (Figure 4B). This might be because the blanched tissue is more conducive to microbial fermentation and the metabolism production of lactic acid (Tae-Kyung et al., 2019).

The pH values of bamboo shoots subject to different vacuum pressure show a downward trend with the extension of pickling days, except for the 2nd day (Figure 4C). There was no significant difference existing in pH values at different vacuum pressures, demonstrating that adjusting vacuum pressure was not beneficial to improving the sourness of the bamboo shoots samples. Similar trends were also observed in different pulsation frequency ratio groups as shown in Figure 4D. The same conclusions were revealed by Rao et al. (2019) on Sichuan paocai and found that the brine samples in all groups presented a continuous decline in pH value, reducing from 7.0 to 3.0.

### Color analysis

The effects of different pickling experimental conditions on the color attributes of bamboo shoots on the 10th day are shown in Table 3. The values of ΔE^*^ are in the range of 14.95–38.88 and the L^*^ values vary from 31.27 to 54.32. The minimum ΔE^*^ value of 14.95 and highest L^*^ value of 54.32 are both obtained in the salt solution concentration of 3% in the traditional pickling group with blanching. No significant differences were found in the color values (L^*^, a^*^, and b^*^) of bamboo shoot samples under different salt solution concentrations, vacuum pressure, and pulsation frequency ratio conditions (*p* > 0.05). It can be seen that the standard deviations of the color values are relatively large. That might be because bamboo shoots produce organic acid during the fermentation process, which promotes the conversion of chlorophyll into pheophytin. Therefore, the color of the bamboo shoots becomes lighter and pickling makes the shoots translucent. Chung et al. (2008) studied the color protection of bamboo shoots and also found that the standard deviation of bamboo shoots was greater than the precision of the machine, which may be due to the refraction and diffraction of light and therefore resulted in a large deviation of the color value.

**Table 3:** Colors of sour bamboo shoots under different PVPP parameters.

**Technical parameters**	L*	a*	b*	ΔE*
0%	41.02, 6.43^bcd^	−9.68, 2.76^abc^	21.91, 9.99^d^	38.88, 8.55^a^
3%	34.89, 4.24^cd^	−8.10, 1.44^ab^	24.46, 7.64^cd^	34.62, 6.52^ab^
6%	47.44, 3.79^abc^	−7.54, 1.05^a^	35.90, 7.38^abcd^	20.48, 3.31^cdef^
9%	35.56, 13.02^cd^	−7.02, 3.04^a^	27.56, 9.38^bcd^	33.31, 11.87^abc^
Blanched 0%	49.98, 0.25^ab^	−7.78, 0.86^ab^	44.90, 1.86^a^	14.97, 4.66^f^
Blanched 3%	54.32, 2.20^a^	−8.65, 1.51^ab^	36.42, 9.02^abcd^	14.95, 9.71^f^
Blanched 6%	50.96, 7.60^ab^	−9.06, 1.69^ab^	31.93, 10.93^abcd^	20.08, 11.00^cdef^
Blanched 9%	44.48, 6.94^abc^	−13.01, 1.56^c^	37.96, 4.19^abc^	17.89, 8.61^ef^
20 kPa	35.31, 6.45^cd^	−8.94, 0.83^ab^	45.16, 3.62^a^	29.84, 9.06^abcde^
40 kPa	45.10, 3.96^abc^	−8.29, 1.09^ab^	41.22, 1.83^ab^	18.71, 2.23^def^
60 kPa	35.96, 7.96^cd^	−10.31, 1.87^abc^	29.06, 7.68^bcd^	35.24, 4.78^ab^
80 kPa	40.52, 5.09^bcd^	−8.82, 1.48^ab^	24.93, 4.51^cd^	23.51, 10.85^bcdef^
5:04 min/min	31.27, 8.19^d^	−7.94, 3.47^ab^	38.71, 4.16^abc^	32.63, 6.64^abcd^
10:04 min/min	36.99, 4.16^cd^	−11.60, 1.90^bc^	28.80, 7.19^bcd^	35.28, 1.11^ab^
15:04 min/min	46.95, 8.22^abc^	−8.40, 3.71^ab^	33.83, 11.27^abcd^	25.27, 2.87^abcdef^
20:04 min/min	44.03, 1.84^abc^	−9.64, 0.38^abc^	33.40, 13.36^abcd^	26.90, 3.50^abcdef^

According to the Duncan test, the different letters in the same column reveal significant difference at α = 5% level.

From Table 3, the ΔE^*^ value of the traditional control groups with and without blanching decreased initially and then increased as the concentration of salt in the solution increased from 0 to 9%. The traditional control group with blanching had a relatively better color in comparison with the unblanched group. The lightness L^*^ ranged from 44.48 to 54.32, and ΔE^*^ value varied from 14.95 to 20.08, indicating that the blanching treatment was beneficial for maintaining good color quality. This can be attributed to the inactivation of enzyme activity during hot water blanching which prevents the enzymatic browning reaction (Deng et al., 2017). Meanwhile, the color values (L^*^, a^*^, and b^*^) under PVPP parameters show a fluctuating trend, and no obvious differences were found in terms of color attributes (*p* > 0.05). Reducing the vacuum pressure or adjusting the pulsation frequency ratio would not improve the color quality of bamboo shoots compared to the traditional control group. It might be because the pickling process of bamboo shoots immersed in the saline solution factually isolates the air, limits the main oxidative effect, and therefore weakens the color protection effect of PVPP technology (Fan et al., 2022). Hence, in terms of color protection, blanching treatment is more effective than PVPP for bamboo shoots.

### Crunchiness and chewiness

It can be seen as marinating time increases, the crunchiness values show a fluctuating trend as a whole (Table 4). Ye et al. (2020) obtained similar results during the pickling of Chinese pepper (Paojiao) and no clear trend was observed in terms of hardness and chewiness. There was no obvious difference (*p* > 0.05) in the crunchiness of bamboo shoots under different vacuum pressures and pulsation frequency ratios. The crunchiness of the traditional control group without blanching was relatively smaller than that of blanched control groups and PVPP groups, especially on the 8th day, which gained the smallest value of 0.58 N at the salt solution concentration of 3%.

**Table 4:** Fragility value (N) of sour bamboo shoots during pickling.

	**0 day**	**2 days**	**4 days**	**6 days**	**8 days**	**10 days**
0%	1.03 ± 0.03^ab^	1.06 ± 0.03^d^	1.02 ± 0.01^cd^	1.02 ± 0.02^d^	0.62 ± 0.09^e^	0.68 ± 0.29^b^
3%	1.15 ± 0.03^a^	1.09 ± 0.01^abcd^	1.12 ± 0.03^ab^	1.13 ± 0.01^abc^	0.58 ± 0.09^e^	1.13 ± 0.02^a^
6%	1.13 ± 0.03^a^	1.14 ± 0.01^a^	1.12 ± 0.01^ab^	1.14 ± 0.02^ab^	0.94 ± 0.02^c^	1.15 ± 0.01^a^
9%	1.13 ± 0.01^a^	1.13 ± 0.04^abc^	1.08 ± 0.01^abc^	1.04 ± 0.11^cd^	0.82 ± 0.02^d^	1.13 ± 0.01^a^
Blanched 0%	1.05 ± 0.04^ab^	1.08 ± 0.04^bcd^	1.08 ± 0.05^abc^	1.09 ± 0.03^abcd^	1.00 ± 0.19^bc^	1.00 ± 0.18^a^
Blanched 3%	1.13 ± 0.01^a^	1.12 ± 0.04^abc^	1.12 ± 0.03^ab^	1.05 ± 0.02^bcd^	1.07 ± 0.01^abc^	1.08 ± 0.04^a^
Blanched 6%	0.90 ± 0.06^b^	1.10 ± 0.02^abcd^	1.10 ± 0.02^abc^	1.09 ± 0.02^abcd^	1.08 ± 0.05^ab^	1.07 ± 0.07^a^
Blanched 9%	1.10 ± 0.02^ab^	1.08 ± 0.01^cd^	1.09 ± 0.02^abc^	1.09 ± 0.01^abcd^	1.11 ± 0.03^ab^	1.13 ± 0.03^a^
20 kPa	0.66 ± 0.45^c^	1.12 ± 0.02^abc^	1.11 ± 0.03^abc^	1.12 ± 0.03^abc^	1.10 ± 0.06^ab^	1.09 ± 0.10^a^
40 kPa	1.14 ± 0.02^a^	1.11 ± 0.03^abcd^	1.04 ± 0.13^bcd^	0.91 ± 0.01^e^	1.00 ± 0.11^bc^	1.14 ± 0.01^a^
60 kPa	1.14 ± 0.01^a^	1.12 ± 0.04^abc^	0.96 ± 0.10^d^	1.02 ± 0.13^d^	1.15 ± 0.01^a^	1.00 ± 0.14^a^
80 kPa	1.12 ± 0.01^ab^	1.11 ± 0.03^abcd^	1.11 ± 0.05^abc^	1.10 ± 0.04^abcd^	1.13 ± 0.03^ab^	1.11 ± 0.04^a^
5:04 min/min	1.15 ± 0.01^a^	1.13 ± 0.03^abc^	1.12 ± 0.05^ab^	1.15 ± 0.01^a^	1.13 ± 0.02^ab^	1.14 ± 0.03^a^
10:04 min/min	1.13 ± 0.03^a^	1.14 ± 0.01^ab^	1.09 ± 0.02^abc^	1.14 ± 0.03^ab^	1.12 ± 0.02^ab^	1.15 ± 0.02^a^
15:04 min/min	1.11 ± 0.02^ab^	1.13 ± 0.02^abc^	1.15 ± 0.02^a^	1.10 ± 0.03^abcd^	1.15 ± 0.01^a^	1.15 ± 0.01^a^
20:04 min/min	1.11 ± 0.03^ab^	1.12 ± 0.03^abc^	1.11 ± 0.03^abc^	1.13 ± 0.02^abc^	1.11 ± 0.06^ab^	1.15 ± 0.01^a^

Different letters in the same column reveal significant difference at α = 5% level according to the Duncan test.

**Figure 5:**
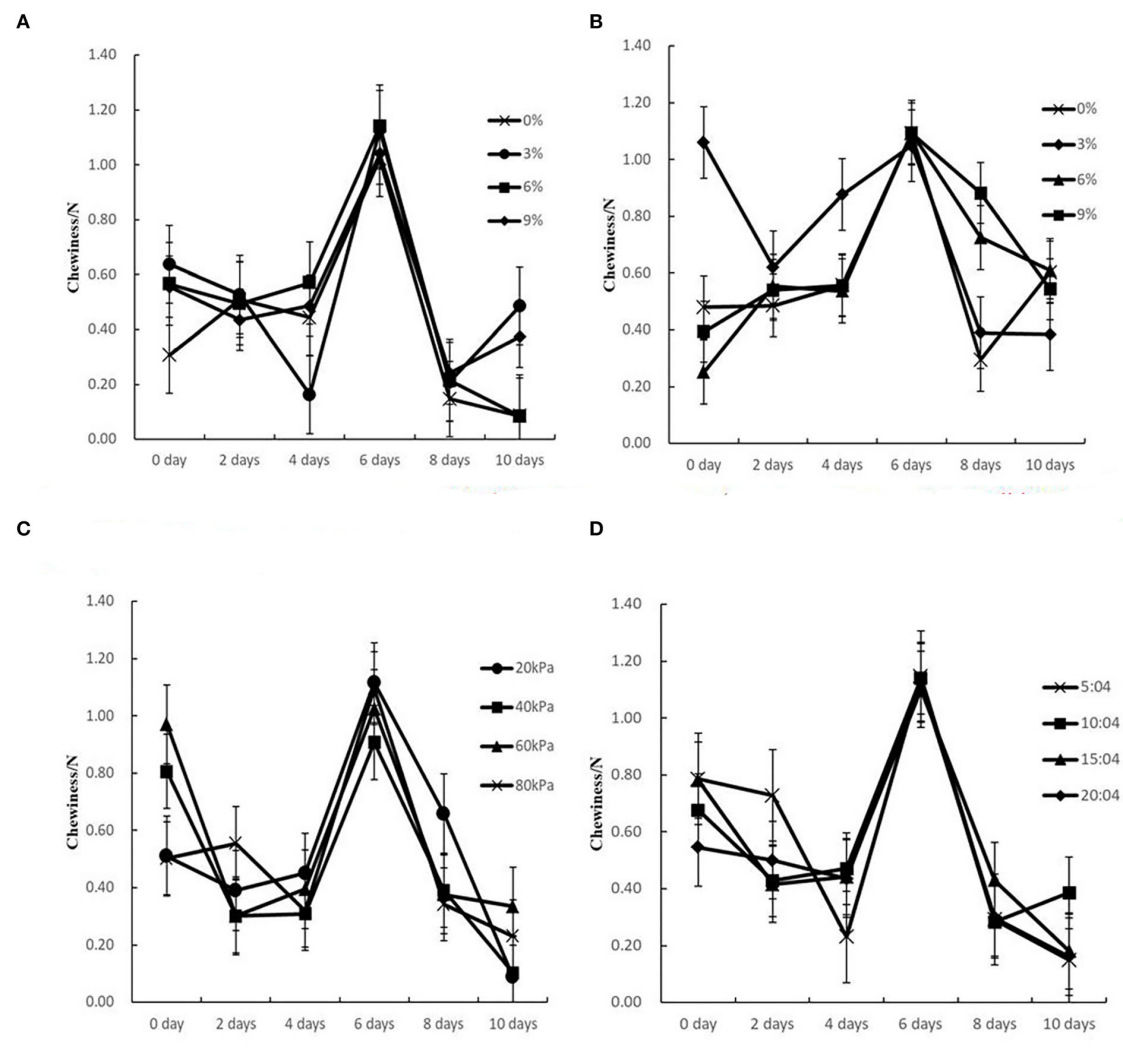
Chewiness kinetic curves of sour bamboo shoots under different technical parameters. **(A)** Traditional control groups without blanching under different salt concentrations, **(B)** Traditional control groups with blanching under different concentrations, **(C)** PVPP groups with different vacuum pressures, **(D)** PVPP groups with different pulsation frequency ratios.

An obvious wave crest is visible in all the chewiness kinetics curves under different pickling parameters (Figure 5). The chewiness values in different PVPP groups generally decreased at first and then increased until the 6th day with the maximum value of 1.15N obtained at the pulsation frequency ratio of 5:4 min/min. As reported in many works, the texture of bamboo shoots shows a significantly decreasing trend during pickling (Zheng et al., 2013), which is different from that of pickled bamboo shoots in this research. The difference may be attributed to the different fermentation processes between PVPP technology and the traditional pickling method.

### LAB content

The LAB contents of bamboo shoots under different PVPP parameters are shown in Table 5. Lactic acid bacterial contents are the predominant microorganisms during the pickling process of bamboo shoots which has a similar change trend to the total aerobes (Luo et al., 2020). It can be seen that the CFU of LAB inside the bamboo shoots rises to begin with and then falls until the 6th day and fluctuates with the extension of the pickling time. It coincides with the findings of Liu et al. (2017) on Sichuan paocai, who noted that the CFU of LAB increases to the maximum value on the 4th day of fermentation. Subsequently, it begins to drop and then increases again from day 7 to day 10. The traditional control group without blanching had a relatively lower LAB content as the salt solution concentration increased from 0 to 9%, while LAB content in the blanched control group first increased and then decreased with the increase in salt concentration. No clear variation rules were found between the vacuum pressure groups and pulsation frequency ratio groups as technical parameters changed. Compared with the traditional pickling methods at the same salt concentration of 6%, bamboo shoots under PVPP conditions obtain relatively more lactic acid bacteria content, which is key for the formation of the sour flavor during the fermentation of bamboo shoots.

**Table 5:** CFU average content of sour bamboo shoots during pickling under different technical parameters (CFU mL^−1^(×10^−5^)).

**Technical parameters**	**0 day**	**3 days**	**6 days**	**9 days**
0%	3.67, 1.15^cd^	181.67, 4.16^c^	121.00, 16.64^a^	191.00, 17.09^a^
3%	4.33, 1.53^cd^	134.33, 12.01^ef^	36.00, 7.00^bcd^	83.67, 12.58^c^
6%	3.00, 1.73^cd^	136.33, 16.65^ef^	17.33, 4.16^e^	29.67, 2.31^fgh^
9%	9.33, 3.21^c^	31.67, 8.50^j^	23.33, 4.93^de^	17.00, 2.65^gh^
Blanched 0%	3.33, 0.58^cd^	73.00, 11.14^i^	40.33, 11.15^bc^	68.67, 19.55^cd^
Blanched 3%	3.67, 1.53^cd^	93.33, 8.50^hi^	49.33, 9.29^b^	34.00, 4.36^efg^
Blanched 6%	0.67, 0.58^d^	117.67, 9.29^fg^	23.00, 6.93^de^	52.67, 11.59^de^
Blanched 9%	0.33, 0.58^d^	42.00, 11.00^j^	29.67, 4.62^cde^	13.33, 1.53^h^
20 kPa	1.33, 0.58^d^	162.67, 15.95^cd^	24.00, 2.65^de^	13.67, 4.73^h^
40 kPa	63.33, 11.59^a^	137.33, 16.62^ef^	23.33, 4.16^de^	79.67, 13.65^c^
60 kPa	5.00, 1.00^cd^	98.67, 4.04^gh^	47.00, 8.66^b^	84.67, 9.50^c^
80 kPa	20.33, 5.51^b^	156.67, 17.01^de^	24.33, 4.04^de^	126.33, 18.93^b^
5:04 min/min	1.00, 1.73^d^	219.00, 21.93^b^	27.00, 2.65^cde^	39.00, 2.00^ef^
10:04 min/min	1.33, 1.53^d^	121.00, 18.73^fg^	17.00, 2.00^e^	78.33, 9.07^c^
15:04 min/min	26.67, 8.14^b^	247.67, 13.58^a^	32.67, 8.08^cd^	66.67, 10.97^cd^
20:04 min/min	4.67, 0.58^cd^	72.67, 1.53^i^	30.33, 6.66^cde^	81.33, 11.37^c^

Different letters in the same column reveal significant difference at α = 5% level according to the Duncan test.

### Residual nitrite content of bamboo shoots

**Figure 6:**
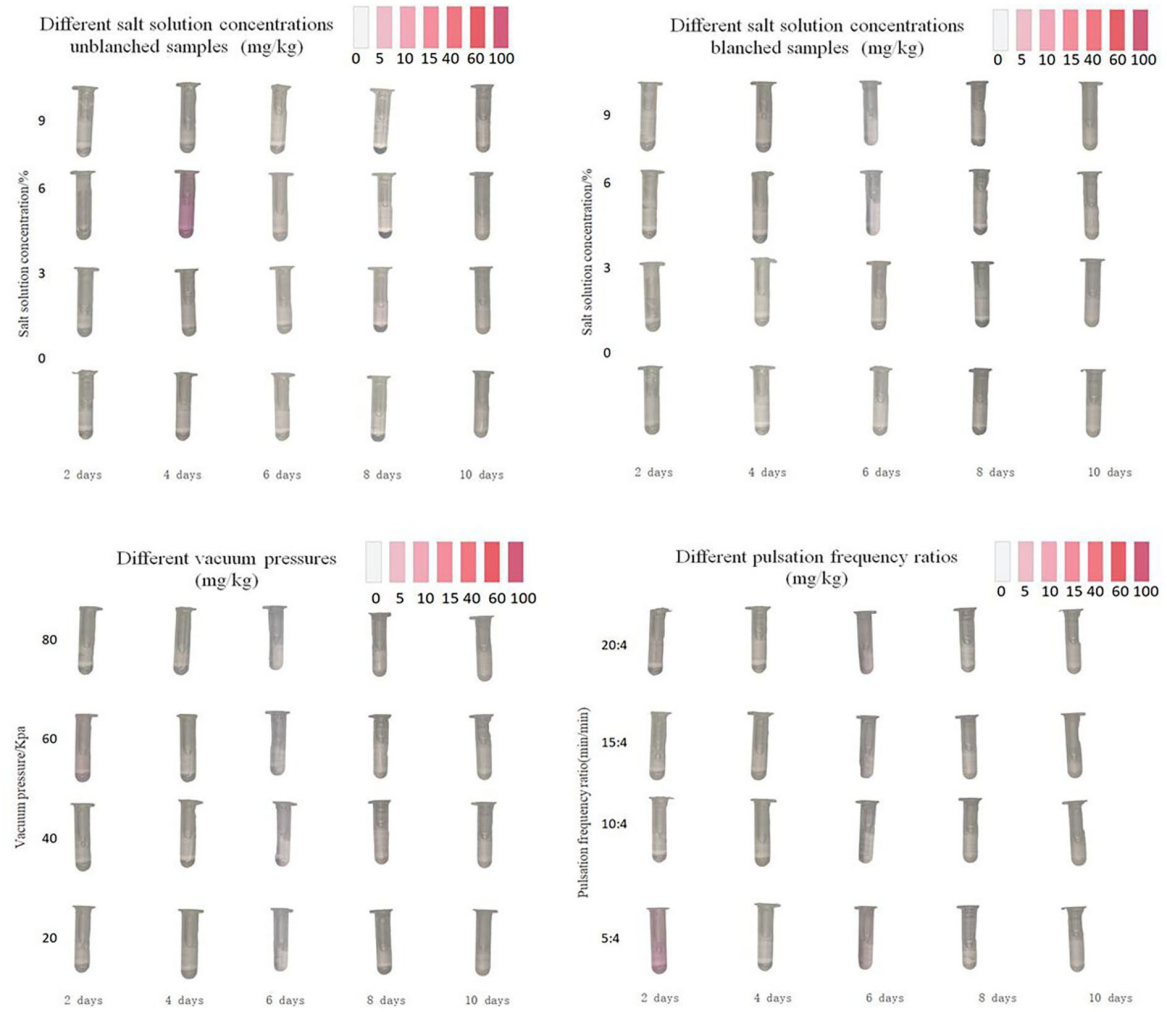
Nitrite content variations of sour bamboo shoots during pickling.

The residual nitrite content of bamboo shoots during pickling is listed in Figure 6. The experimental groups and traditional control groups did not exceed the safety standards (National Standard of the People's Republic of China, 2017) specified by Chinese National Food Safety Standard GB 2762-2017 (<20 mg/kg). Among the whole PVPP experimental groups, the pulsation frequency ratio of 5:04 min/min group gained a relatively larger nitrite content of about 5 mg/kg on the 2nd pickling day, and then the residual nitrite content declined continuously. Meanwhile, the traditional control group without blanching immersed in the salt solution concentration of 6% obtained the highest value of 10 mg/kg on the 4th pickling day and the sample showed an obvious chromogenic reaction. It can be found that the peak value of nitrite content under different technical conditions occurred on different pickling days, while other researchers reveal that marinating time at peak point also depended on the type of pickled samples (Ye et al., 2020). Wu et al. (2015) reported that the residual nitrite content of sauerkraut gains its peak point after 18 pickling days, whereas Yan et al. (2008) and Ji et al. (2009) confirm the nitrite content inside the pickled Chinese cabbage reached the highest value on the 2nd and 3rd days, respectively.

### Overall rating score

Each experimental data was standardized and the weight coefficients of the different quality indices were determined to obtain comprehensive scores, as shown in Table 6. The weight coefficients of salt content, pH value, crunchiness, chewiness, L^*^, and Δ*E* were found to be 0.115, 0.187, 0.050, 0.302, 0.147, and 0.199, respectively.

**Table 6:** Comprehensive score of sour bamboo shoots under different technical parameters.

**No**.	**Technical parameters**	**Salt content (%)**	**pH value**	**Fragility (N)**	**Chewiness (N)**	L*value	**Δ*E***	**Comprehensive score F**
1	0%	0.00	4.68	0.68	0.09	41.02	38.88	0.45
2	3%	2.14	4.19	1.13	0.49	34.89	34.62	0.59
3	6%	3.32	4.37	1.15	0.09	47.44	20.48	0.40
4	9%	5.01	4.14	1.13	0.37	35.56	33.30	0.55
5	Blanched 0%	0.00	4.06	1.00	0.60	49.98	14.97	0.52
6	Blanched 3%	1.98	3.85	1.08	0.38	54.32	14.95	0.44
7	Blanched 6%	3.37	3.70	1.07	0.61	50.96	20.08	0.56
8	Blanched 9%	5.10	3.68	1.13	0.54	44.48	17.89	0.54
9	20 kPa	4.35	3.91	1.09	0.09	35.31	29.84	0.34
10	40 kPa	4.41	4.16	1.14	0.10	45.10	18.71	0.37
11	60 kPa	4.34	3.82	0.99	0.34	35.96	35.24	0.50
12	80 kPa	4.41	3.91	1.11	0.23	40.52	23.51	0.40
13	5:04 min/min	4.41	4.09	1.14	0.15	31.27	32.63	0.41
14	10:04 min/min	4.49	3.86	1.15	0.39	36.99	35.28	0.60
15	15:04 min/min	4.61	4.08	1.15	0.18	46.95	25.27	0.47
16	20:04 min/min	4.78	3.97	1.15	0.16	44.03	26.90	0.44

According to the Duncan test, the different letters in the same column represent significant difference at α = 5% level.

As the salt solution concentration increased from 0 to 9%, the comprehensive scores *F*, ranging from 0.40 to 0.59, show a fluctuating trend. The total scores of the control group with blanching treatment are overall near the traditional control groups without blanching and vary from 0.44 to 0.56. Meanwhile, the *F* value increased initially and dropped subsequently with the increase of vacuum pressures and pulsation frequency ratios. The optimal pickling parameter for bamboo shoots under PVPP technology (highest *F*-value of 0.60) was obtained at the pulsation frequency ratio of 10:04 min/min with a vacuum pressure of 60 kPa and a salt solution concentration of 6%.

## Conclusion

The final salt content of bamboo shoots in the PVPP experimental groups, varying from 4.40 to 4.97%, was 34.1% higher than the traditional control groups, suggesting that PVPP technology could enhance mass transfer efficiency significantly. Similarly, the uniformity of salt distribution inside the bamboo shoots under PVPP technology showed a better performance. The pH value gradually dropped from 5.96 to 3.70 as the marination duration was prolonged. The blanched control group obtained a more sour quality compared to the traditional unblanched group with the same marinating time. No significant differences were found in the color values (L^*^, a^*^, and b^*^) and the crunchiness of the bamboo shoot under different salt solution concentrations, vacuum pressure, and pulsation frequency ratio conditions. The minimum ΔE^*^ of 14.95 and highest L^*^ of 54.32 were both obtained with the salt solution concentration of 3% in the traditional control group with blanching. An obvious wave crest existed in all the chewiness kinetics curves and the maximum value of 1.15 N was found at the pulsation frequency ratio of 5:4 min/min on the 6th pickling day.

The CFU of LAB in the traditional control group without blanching had a relatively lower population as the salt solution concentration increased from 0 to 9%. All the PVPP experimental groups and traditional control groups met the safety standards with regard to nitrate content (<20 mg/kg). As the vacuum pressure and pulsation frequency ratio increased, *the F*-value increased initially and declined subsequently. The PVPP technology can safely replace the traditional method with better quality performance. The optimal pickling parameter for bamboo shoots under PVPP technology was obtained at the pulsation frequency ratio of 10:04 min/min, a vacuum pressure of 60 kPa, and a salt solution concentration of 6% with the highest *F*-value of 0.60.

## Data availability statement

The original contributions presented in the study are included in the article/supplementary material, further inquiries can be directed to the corresponding author.

## Author contributions

J-WD: conceptualization, funding acquisition, project administration, and writing review and editing. QZ: methodology, supervision, and writing—original draft. ML: investigation. L-JL, S-XL, and Y-LL: methodology. L-JX: validation. Y-WL: conceptualization. P-FY: writing review and editing. Y-PZ: software. WQ: project administration supervision. All authors contributed to the article and approved the submitted version.

## Funding

This research was funded by the National Natural Science Foundation of China (No. 32101974) and the Science Technology Project of Sichuan (No. 2020JDRC0066).

## Conflict of interest

The authors declare that the research was conducted in the absence of any commercial or financial relationships that could be construed as a potential conflict of interest.

## Publisher's note

All claims expressed in this article are solely those of the authors and do not necessarily represent those of their affiliated organizations, or those of the publisher, the editors and the reviewers. Any product that may be evaluated in this article, or claim that may be made by its manufacturer, is not guaranteed or endorsed by the publisher.
